# Congruency of Separable Affix Verb Combinations Is Linearly Indexed by the N400

**DOI:** 10.3389/fnhum.2018.00219

**Published:** 2018-05-28

**Authors:** Jeff Hanna, Friedemann Pulvermüller

**Affiliations:** ^1^Department of Neurosurgery, University Hospital Erlangen, Erlangen, Germany; ^2^Department of Philosophy and Humanities, Institute for German and Dutch Philology, Freie Universität Berlin, Berlin, Germany; ^3^Berlin School of Mind and Brain, Humboldt-Universität zu Berlin, Berlin, Germany

**Keywords:** N400, ERP, morphosyntax, semantics, linear mixed models

## Abstract

Separable affix verbs consist of a stem and a derivational affix, which, in some languages can appear together or in discontinuous, distributed form, e.g., German “aufgreifen” and “greifen … auf” [“up-pick(ing)” and “pick … up”]. Certain stems can combine with only certain affixes. However, many such combinations are evaluated not as clearly correct or incorrect, but frequently take an intermediate status with participants rating them ambiguously. Here, we mapped brain responses to combinations of verb stems and affixes realized in short sentences, including more and less common particle verbs, borderline acceptable combinations and clear violations. Event-related potential responses to discontinuous particle verbs were obtained for five affixes re-combined with 10 verb stems, situated within short, German sentences, i.e., “sie <stem>en es <affix>,” English: “they <stem> it <affix>.” The congruity of combinations was assessed both with behavioral ratings of the stimuli and corpus-derived probability measures. The size of a frontal N400 correlated with the degree of incongruency between stem and affix, as assessed by both measures. Behavioral ratings performed better than corpus-derived measures in predicting N400 magnitudes, and a combined model performed best of all. No evidence for a discrete, right/wrong effect was found. We discuss methodological implications and integrate the results into past research on the N400 and neurophysiological studies on separable-affix verbs, generally.

## Introduction

When participants hear a sentence while under neurophysiological investigation, they often produce a negative deflection in the event-related potential (ERP) waveform peaking approximately 400 ms after the onset of each word in the sentence, known as the N400 (Kutas and Federmeier, 2011). Crucially, the amplitude of the N400 has been found to increase reliably with the degree to which the word does not fit the semantic expectations generated by previous words in the sentence (Petten, 1993, for review). Until recently, these investigations have been operationalized mostly using categorical designs: brain responses to stimuli are grouped and averaged into categories such as, e.g., highly anomalous, less anomalous, not anomalous, etc., and differences between these averages are analyzed using standard statistical tests for comparing means, such as ANOVAs or *t*-tests.

More recently, however, semantic anomalies or other variables of interest have been operationalized continuously, rather than categorically. Generally, this involves averaging across participants, rather than items, and regressing the desired linguistic properties of the items against ERP/F values (King and Kutas, 1998; Hauk et al., 2006, 2009). Focussing on the N400 in particular, correlational studies have found reliable, continuous relationships between N400 amplitude and orthographic neighborhood, lexical association (Laszlo and Federmeier, 2011) as well as semantic properties (Van Petten, 2014).

An alternative paradigm uses linear mixed effects (LMEs) models for ERP/F data. These are a highly flexible application of the general linear model, where any number or combination of random effects (i.e., participants and items) or fixed effects (experimental manipulations) may be specified at will, either as categorical or continuous variables. In this way, parameters can be estimated for a linear relationship between dependent and independent variables in conjunction with individual parameters for, e.g., each participant, stimulus, or stimulus property. In contrast with typical ERP/F regression approaches, where ERP/Fs are averaged across participants and then regressed against independent variables, each participant retains his/her own ERP/Fs. This is a more powerful statistical model than across-participants averaging, as the latter excludes information about the variance across participants.

Linear mixed effect models have been used to observe relationships between the N400 and continuous variables such as absolute and relative word frequency, word position, predictability and interactions thereof, for single-trial EEG responses to natural linguistic material (Dambacher et al., 2006; Alday et al., 2017). Another study used LME to regress surprisal – the degree to which a word is unexpected given the preceding context – against many of the typical language ERP components (Frank et al., 2015), and found that only the N400 component correlated with surprisal. Furthermore, surprisal was derived from language corpus data with a variety of algorithms, including Markov chains, recursive neural networks, and phrase structure grammars using the words’ parts of speech. The more accurate an algorithm performed, the better its surprisal measures fit the N400 values. These results highlight another advantage of using LMEs over comparison of means: the efficacy of multiple competing models/hypotheses can be easily compared to each other in a clear, principled way.

This ability to directly compare models opens up a range of interesting new possibilities in ERP research. Typically, studies collect a single measure with which to categorize stimuli, e.g., a behavioral measure such as cloze probability or reaction latencies, or more recently, information culled from language corpora. Now, however, we can compare multiple measures to determine the factor, or combination of factors, to which ERP modulations are maximally sensitive. To this end, we have sought to compare ERP responses to material whose linguistic properties are assessed with both a corpus-derived statistic, namely pointwise mutual information (PMI), and acceptability ratings. While one expects behavioral and corpus-based measures to correlate strongly with each other, they must also diverge in other aspects (Smith and Levy, 2011), caused perhaps by, e.g., meta-linguistic reasoning or other task-related factors immanent to a behavioral measure, or by, e.g., larger sampling error in the corpus measure. Determining how these variable differently relate to ERPs could not only provide methodological guidance on which measure(s) to choose for future experiments, but also better distinguish underlying cognitive mechanisms related to ERPs, in the case that the measures purportedly index distinct processes.

The linguistic phenomenon we have chosen to investigate is the separable affix verb in German. These are verbs which consist of an affix and a stem that can be realized in an utterance either contiguously or separate from each other depending on the grammatical context. For example the word “abholen” (Eng. “pick up”) consists of a prepositional affix “ab” and a stem “holen,” and could be expressed in a sentence as, e.g., “Ich muss ihn abholen” (Eng. “I have to pick him up.”) or “Ich hole ihn ab” (Eng. “I’m picking him up.”) Certain stems can combine with certain affixes, but others not, e.g., “abholen” is acceptable, “anholen” is not; “anpflanzen” is acceptable, “abpflanzen” is not. These are clear examples of correct and incorrect combinations of stem and affix, but many cases are not so simple. Native speakers in fact disagree on the lexical status of many separable affix verbs, or a single native speaker, when given the option to do so, will often classify a separable-affix verb as having ambiguous lexical status, sounding neither entirely wrong nor right. Anecdotally, we have often observed a native speaker assert that a stem-affix pair is not a word, and then change his or her mind upon a few seconds reflection or consultation with another native speaker.

It seems well-motivated then to investigate brain responses to separable-affix verbs, with incongruity operationalized as a continuous variable. Studying brain responses to separable affix verbs also provides a methodological advantage in that there are a limited number of affixes. Therefore the variance in the neurophysiological signal due to acoustic differences is significantly reduced. In fact, in this particular experiment, the same recording of the affix is used in all combinations, rendering acoustic and phonemic variance null across stems. We have employed a full combination of 10 common stems and the five most common affixes. These 50 possible combinations, according to our two assessments, cover a wide spectrum of incongruity, from completely unacceptable, to ambiguous, to completely acceptable. A previous study we conducted with separable-affix pairs, using the linguistic MMN paradigm (Pulvermüller and Shtyrov, 2006) found – in addition to the expected MMN enhancement for correct combinations – a late negative component for incorrect combinations of stem and affix, which we interpreted as an N400 (Hanna et al., 2017).

Therefore we may expect more incongruous pairings of stem and affix to produce stronger N400s. The critical question is whether the N400 would vary continuously with incongruity, as the relatively limited number of items used in Hanna et al. (2017) were not sufficient to make such an inference. Though this study did not use an MMN paradigm like Hanna et al. (2017), the N200 time window could nevertheless once again produce a lexical effect, i.e., more incongruent pairings produce weaker peaks than congruent pairings. In addition to these, we will also examine the N100 peak as an exploratory measure. The relationship between incongruity and ERP peaks was assessed with acceptability ratings, corpus-derived PMI, and the combination of these two, though we make no predictions of how they will perform in relation to each other.

## Materials and Methods

### Participants

Data were recorded from a total of 35 participants. One was excluded on account of technical difficulties during the recording, as well as a further two who produced excessively noisy data, leaving a total of 32 participants (13 males) in the analysis. All participants were right-handed, as confirmed using the Edinburgh Handedness Inventory (Oldfield, 1971), monolingual native speakers of German, and did not report any linguistic, psychiatric, or neurological disorders. Participant ages ranged from 18 to 35, with mean of 25.6 and standard deviation of 4. All participants were recruited from the student/post-graduate population of the Free University Berlin. The experiments were performed with the approval of the Ethics committee of the Charité Universitätsmedizin, Campus Benjamin Franklin, Berlin, and participants gave informed, written consent.

### Design

The stimuli in all cases consisted of a single, short German sentence with a separable-affix verb: “sie <stem>en es<affix>,” English: “they <stem> it <affix>.” Ten possible stems could occupy the <stem>slot, and five possible affixes for the <affix>slot. These five affixes were chosen on the basis that they are the most common in German. The stems were chosen in order to ensure a similar distribution of congruency across the prefixes (see **Figure 3**). All possible combinations were used, making 50 conditions in total. Each of these conditions was presented 30 times for a total of 1500 presentations. Stimuli occurred pseudo-randomly, with a stimulus-onset asynchrony of 2.2 s. The experiment was split into two blocks, with block priority counterbalanced across participants.

The behavioral, sentence-rating experiment consisted of the exact stimuli used in the EEG experiment. The sentence was first presented auditorially, and then displayed orthographically on the screen, and participants were asked to use a button-box to rate the acceptability of the sentence on a scale of 1 to 5.

### Stimuli

**FIGURE 1:**
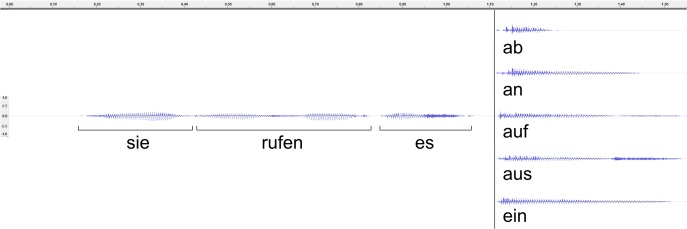
Acoustic waveforms of one of 10 possible stems (“reden”) and its combination with the five possible stems. Time is marked in seconds.

A female, native German speaker was recorded pronouncing the sentence, “sie <stem>en es <affix>” with various stem-affix combinations, several times each in a sound-proof chamber. In order to avoid biasing the pronunciation, affixes were recorded with stems that would not occur in the experiment, and vice versa. The repetitions which had the same intonation were selected, and stems and affixes were extracted from their contexts. These were then combined to make the desired stimulus combinations. When combining stems and affixes, care was taken that the affix began at the same time, in this case 1116 ms after stimulus onset. Finally, sound energy was normalized at -5 db. All sound editing was performed with Audacity (2018) 2.0.3^1^. For waveforms of an example stem and affixes, see **Figure 1**.

### Assessment of Stem-Affix Incongruity

**FIGURE 2:**
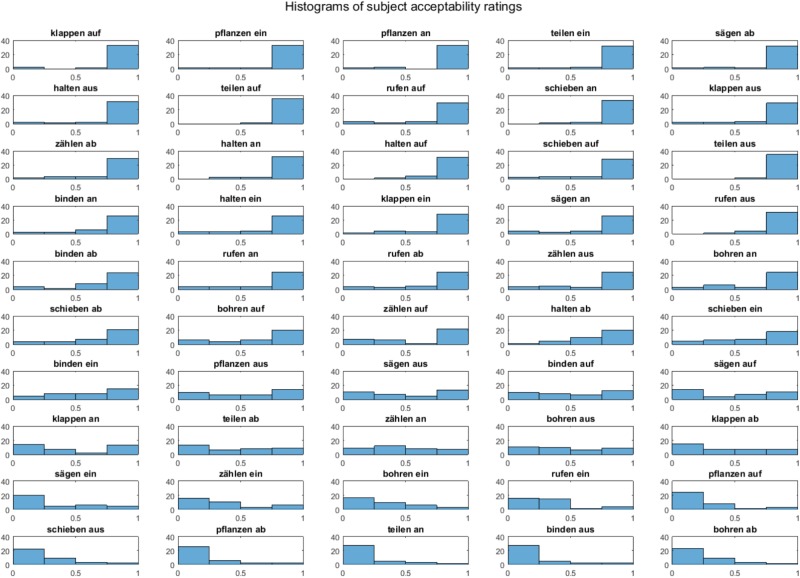
Histograms of participants’ acceptability responses to stem-affix combinations on a scale of 1 to 5. Responses were normalized to 0 to 1, with 1 indicating most acceptable. Y axis indicates the amount of participants.

Information about how well a given stem and affix fit together came from two sources: (1) the acceptability ratings from the behavioral experiment described above, and (2) frequency of occurrence in a German language corpus. Acceptability ratings for a given word were calculated by taking the skewness of the distribution of ratings across participants (see **Figure 2**). Frequencies of occurrence were taken from the DE2014 COW corpus (Schäfer and Bildhauer, 2013^2^) by combining occurrences of a given separable-affix verb in participial (e.g., anrufen- > angerufen) and infinitival (e.g., anrufen- > anzurufen) forms. The reason for this is that, in these forms, both stem and affix are forced to occur within the same word, and can therefore be reliably counted in a corpus search. The overall frequency of any given stem alone, and any given affix alone were also pulled from the corpus. These three measures were then used to calculate the PMI of a given separable-affix verb:

$$\log_{2}\frac{p(a,\;b)}{p_{x}(a)p_{y}(b)}$$

where p(a,b) is the probability of occurrence for a given separable-affix verb, p_x_(a) is the probability of the stem occurring alone, and p_y_(b) is the probability of the affix occurring alone. Lower values indicate higher incongruity between stem and affix. PMI values were then shifted positive and natural log transformed, henceforth abbreviated as log transformed pointwise mutual information (lPMI).

**FIGURE 3:**
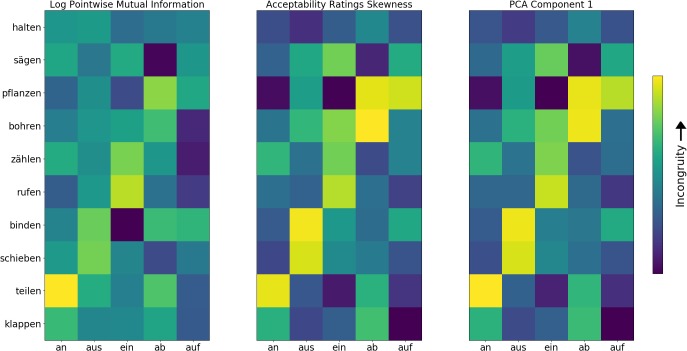
Congruency of stem-affix combinations for all stimuli according to log transformed pointwise mutual information (lPMI), acceptability ratings skewness (ARS), and the first principal component, which summarizes the common variance along the former two. Each square represents the congruency value of a given stem and affix combination. Yellow represents incongruity and blue congruity.

Log transformed pointwise mutual information and acceptability ratings skewness (ARS) correlated highly with one another (*r* = 0.64). In order to further check the validity of the two measures, as well as to combine them into a unified measure and factor out the noise inherent to each individual measure, a principle component analysis was carried out, with lPMI and ARS as the two variables. It was found that the first component explained 90% of the variance. Therefore, our unified incongruity measure was the PCA transformation of ARS and lPMI along this first component. The eigenvector of the first component was [0.33, 0.95] for lPMI and ARS respectively, indicating that the latter is rather more influential in the formation of the first component than the former. Summaries of the lPMI, ARS, and first component transformation of the former two are found in **Figure 3**.

### Procedure

Participants were seated in a comfortable chair facing a monitor, through which they watched a silent distractor movie with no linguistic content. They were instructed that they should ignore the acoustic stimuli, and may simply relax and watch the film. Stimuli were presented binaurally through high-quality headphones. There was a short break between the blocks. The experiment lasted in total about 55 min, excluding preparation. After removal of the cap and washing their hair, each participant took part in the behavioral experiment, which lasted another 5–10 min.

### Recording

Electroencephalography data were recorded with 64 active electrodes (actiCAP system, BrainProducts, Gilching, Germany). Scalp electrodes followed the actiCAP 64 channel arrangement, but with the following modifications: the electrodes in the PO9 and PO10 position were reassigned as EOG channels. The reference was moved from the FCz position to the nose tip, and the electrode occupying the Oz position was reassigned to the empty FCz position. These posterior electrodes were chosen because, in addition to usually being noisy, they have not generally shown interesting responses to auditory linguistic input in past experiments. Data were online band-pass filtered (0.1–250 Hz) and sampled at 500 Hz. Recordings were taken in an electrically and acoustically shielded chamber.

### Preprocessing

Preprocessing was done in MNE-Python 0.14 (Gramfort et al., 2013, 2014). Data were first IIR band-pass filtered at 0.45–40 Hz. Bad channels were algorithmically identified and removed using the ANOAR package (^3^see **Appendix**). Non-ocular artifacts were then identified and removed using ANOAR. Independent component analysis (ICA) was used to separate the data into 32 components, and those components correlating with the ocular signal were removed (MNE: ica.find_bads_eog, threshold = 3). Finally, remaining artifacts were removed using ANOAR, and missing channels were interpolated. Data were epoched from 200 ms before stimulus onset to 2000 ms after onset. No explicit baseline subtraction was performed; low frequency noise was instead eliminated through the 0.45 Hz high-pass filter, which matches the length of the epoch. This approach can be well-suited to experiments with streams of speech without long pauses (Widmann et al., 2015).

Statistics were performed using the lme4 LMEs package version 1.2.10 (Bates et al., 2015) in R version 3.4.1 (R Core Team, 2017), and PCA was performed with scikit-learn 0.18.1 (Pedregosa et al., 2011).

## Results

**FIGURE 4:**
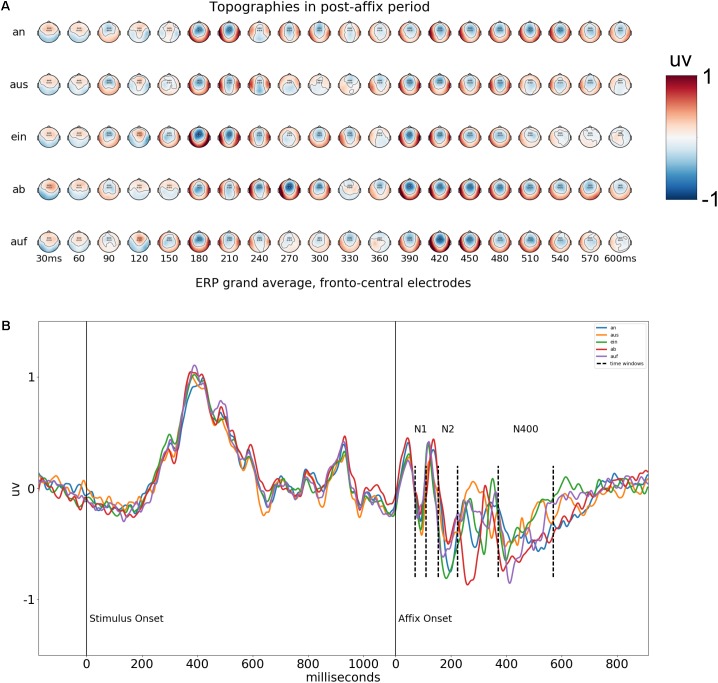
**(A)** Voltage topographies after presentation of the affix. Times are in relation to onset of the affix. The six electrodes used for the average waveform are marked with circles on the topographies. **(B)** Average waveform of the six fronto-central electrodes across the entire epoch, for each possible affix. Time windows used for analysis are marked with the dashed vertical lines.

The grand average topographies in **Figure 4A** show that in the post-affix period, there are a series of negative deflections focussed in the fronto-central electrodes. We selected six electrodes at the center of these deflections, Fz, F1, F2, FCz, FC1, and FC2 and used the average of them for further analysis of the grand average waveforms. The results of this average can be found in **Figure 4B**. The negative deflections form a tri-phasic response, which we interpret as an N1-N2-N400 complex. Average voltages were calculated for each of the 50 conditions, for each participant, across the six frontal electrodes, at the time-windows corresponding to each of these peaks, at 70–90, 154–224, and 370–570 ms in post-affix time, also indicated graphically in **Figure 4B**.

**FIGURE 5:**
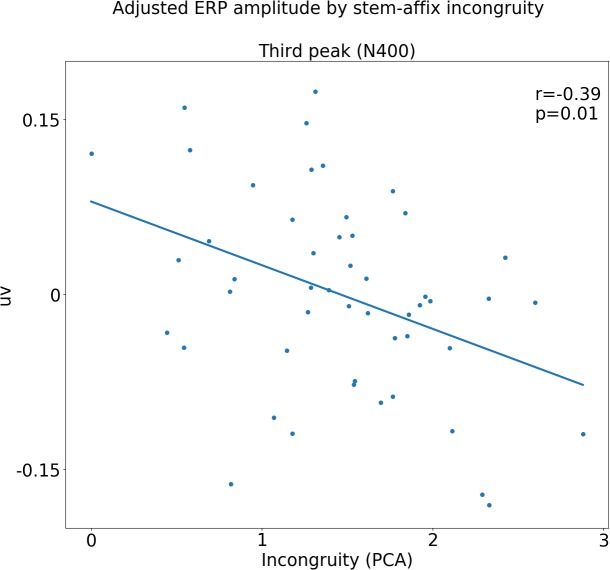
Event-related potential (ERP) amplitude in third time window plotted against incongruity for each affix, with estimated variance due to participant and affix factored out. The upper-right corner shows the results of a linear regression of these adjusted ERP amplitudes against the first principal component.

The relationship between stem-affix incongruency and voltage potential in the N400 time-window was first ascertained using a LMEs analysis as follows: first, the null-model was constructed, containing PARTICIPANT and AFFIX as random, categorical effects. Then the alternate model was constructed, which was the same as the null model, except it also contained INCONGRUENCY as a fixed, continuous effect. The likelihood of the models given the observed data was compared using the Likelihood Ratio Test. The full model was significantly more likely to explain the data than the null model [χ(1) = 7.55, *p* = 0.005], indicating that INCONGRUENCY adds significant explanatory power. Confidence intervals (95%) for the alternate model show that the estimated slope parameter for INCONGRUENCY lies between -0.01 and -0.017 uv, i.e., amplitudes in the N400 time-window become more negative as incongruency between stem and affix increases. To test for differential INCONGRUENCY effects across affixes, an interaction model was built, which was the same as the alternate model, except it also contained separate slope parameters for INCONGRUENCY for each level of AFFIX. The interaction model did not add significant explanatory power in relation to the alternate model [χ(2) = 0.86, *p* = 0.65], suggesting the INCONGRUENCY trend is likely to be uniform across affixes. **Figure 5** shows a scatter plot of the residuals of the null model, i.e., the data with the estimated variance from participants and affixes removed, plotted against incongruity, where the linear relationship can be clearly seen.

The same analysis was then applied to the other time-windows. We found no difference at all in explanatory power of the alternate model over the null model in either the N1 time-window [χ(1) = 0.16, *p* = 0.69] or the N2 time-window [χ(1) = 0.03, *p* = 0.86]. Two further analyses were carried out on the N400 time window, using (1) lPMI or (2) ARS as the INCONGRUENCY measure, rather than PCA component 1. Both measures also produced significant results, albeit less so than PCA component 1 did [lPMI: χ(1) = 6.48, *p* = 0.01; ARS: χ(1) = 7.52, *p* = 0.006]. Finally, a model was built with both lPMI and ARS as separate INCONGRUENCY measures. This model did not improve upon either the lPMI alone model [χ(1) = 0.977, *p* = 0.32] or the ARS alone model [χ(1) = 2.01, *p* = 0.155].

## Discussion

This research was concerned with two questions: the nature of ERP responses to separable-affix verbs of continuously varying incongruency, and the reliability of behavioral and corpus-based measures as correlates of these ERP responses. We found that more incongruent stem-affix pairs produced stronger negative potentials in the later time window, (370–570 ms after affix onset), and this relationship appeared to be fully continuous. There does not appear to be any such relationship in the earlier time windows. Further, both types of measures produced reliable ERP correlations, though the ARS model performed better than the lPMI model, and the two combined using principal component analysis performed best of all. There are three points to discuss in light of these results: (1) potential reasons for the different performance among the measures, (2) the relation of these results to the general N400 literature, and (3) the relation of these results to those of the previous separable affix verb study (Hanna et al., 2017).

### Differing Performance of Measures

There are a few plausible explanations for the fact that the ARS model performed better than the lPMI model. First, it could be that the meta-linguistic aspects of the behavioral task are related to the same processes that produce the N400. Second, the behavioral task requested a judgment on the same stimuli that occurred in the experiment, which included not only the stem and affix, but also the intervening object pronoun “es” as well as the initial subject pronoun “sie.” While we took care that all congruent stem-affix pairs could occur in typical language with these pronouns, it is possible that judgments partially reflect an impression on the whole sentence, which could better correlate with N400 amplitude. Finally, it may simply be a consequence of increased sampling error in the corpus measurements. There are two main sources of noise specific to corpus data. First, even very large corpora are a limited and usually biased sample of the language. Second, the culling procedures for finding instances of the desired phenomenon always contain a degree of error. These two sources of sampling error are likely to produce noisy measures, which would then correlate less reliably with other measures.

The pattern of results indicates that increased sampling error for lPMI is likely the reason for its comparatively worse performance. The key to this argument is the fact that the combined PCA measure performed better than all other models, and that the model with both ASR and lPMI as independent fixed effects did not improve on either the ASR alone or the lPMI alone model. PCA is dimension reducing procedure, which isolates what is common among variables. On the other hand, coefficient estimation in a general linear model maximizes the unique contributions of variables. If the ARS contained unique information, such as, e.g., meta-linguistic cognition or whole sentence assessment, we would have expected that the model with both ASR and lPMI would improve on the lPMI alone model. This was not the case. Rather, if mere sampling error is the cause, we would expect the result we found, namely that the combined PCA model performed best. This is because PCA would extract common signal from both variables, while leaving behind a good deal of noise immanent to both measures, though especially from the noisier one (lPMI). Therefore we suggest that the N400 indeed correlates with what is common to the ARS and lPMI measures: the incongruity of stem and affix pairs, reflected in the former by lower acceptability ratings and in the latter by lower occurrence probability in relation to its component parts. On a methodological level, we may recommend on the basis of these results that corpus derived measures can be an adequate correlate for at least some ERP signals, though behavioral measures may prove superior, and in some cases worth the extra cost of acquisition.

### The N400

As in Hanna et al. (2017), we have interpreted the incongruity-sensitive late time window as an N400, though there are some key morphological difference between our result and a canonical N400. First, the component we found is substantially weaker than that of typical N400s. This however is likely to be a simple consequence of the fact that there were only five potential affixes in the experiment, and the N400 is known to attenuate with repetition (Petten et al., 1991). Second, our late component had a frontal topography in contrast to the typical parietal topography. Frontal N400s (FN400) are in fact well-known in the literature. Initially, the FN400 was thought to reflect familiarity (e.g., having seen an item previously in a behaviorally relevant task) (Curran, 2000), however more recent research has questioned whether there is any functional difference between FN400s and N400s (Kutas and Federmeier, 2011; Voss and Federmeier, 2011), so firm conclusions over the meaning of the component’s topography cannot presently be drawn. One contribution this study makes to the general body of N400 results is the use of closed-class words, namely the prepositions serving as affixes, whereas N400 research has overwhelmingly focussed on open-class words. Earlier ERP work has shown reduced N400s and altered early responses for function words compared to matched content words (Neville et al., 1992; Pulvermüller and Schumann, 1994). This, too, may contribute to a potential explanation for the different morphology of our N400 effect, as both the frequency and semantic structure of prepositions is rather different than that of open-class words.

The precise cognitive/linguistic mechanisms giving rise to the N400 cannot be clarified here, as our data are consistent with two distinct explanations. We could see the N400 as an index of semantic unification between the stem and the affix, or we could see the stem and affix as a whole form or a single word, in which case the N400 may also be reflecting the simple frequency of occurrence of this whole form (for a review of linguistic phenomena associated with the N400 see Kutas and Federmeier, 2011). Further research could tease these factors apart. Other promising avenues for ERPs and linear mixed models include quantifying linguistic properties of the verb (e.g., animacy and transitivity) and determining their contribution to the neurophysiological signal, rather than the more generic rating/probability measures we have taken here.

### Relationship to Earlier Work on Early Linguistic ERP Responses, Including the MMN

We had examined ERP responses to German separable affix verbs in a previous study using the linguistic MMN paradigm. There, incongruity was operationalized as a dichotomous variable, and the goal was to ascertain on the basis of the direction of the MMN deflection whether separable affix verbs were processed as whole forms or as two units combined by a process (for an explanation of this paradigm, see also Cappelle et al., 2010; Hanna and Pulvermüller, 2014). As such, the MMN deflection amplitude was the main object of investigation in that study, and the later enhanced N400 for incongruent stem-affix pairs was entirely unexpected. Here, we have observed once again such an N400, and in addition we have established that it varies continuously with stem-affix incongruity. One difference, however, between the two experiments is that no early effect (150–200 ms) was found in the present study. Even though there was a negative deflection in this time period, with a similar topography to the mismatch negativity, it did not demonstrate any sensitivity to the incongruity of the stem-affix pairs. This would suggest that the increased MMN amplitude generally found for lexical items (Korpilahti et al., 2001; Pulvermüller et al., 2001, 2004; Kujala et al., 2002; Shtyrov and Pulvermüller, 2002; Endrass et al., 2004; Pettigrew et al., 2004; Shtyrov et al., 2005, 2010) may occur only with the use of MMN experimental paradigms. On the other hand MacGregor et al. (2012) did find lexical enhancements at 50–80, 110–170, and 320–520 ms in with a non-MMN paradigm. It is not possible to say definitively here what the reason may be for this difference, but it could have to do with the different acoustic properties of the stimuli. MacGregor et al. (2012) exclusively used linguistic items with initial plosives at the critical word recognition point, whereas in our experiment critical items (the affixes) began with vowels. The latter produce comparatively weaker neurophysiological signals, which may not have brought the earlier lexicality effects to a sufficient strength to be observed. With the use of an MMN paradigm, however, signals are also increased on account of the unexpectedness of the stimuli, which would explain why we were able to observe early lexical effects for the same, vowel-initial stimuli in Hanna et al. (2017). In summary, it may be that early lexical enhancements are weak in the neurophysiological signal, and need to be enhanced in order to bring them to an observable threshold, whether from, e.g., an MMN paradigm, or through the use of highly acoustically salient stimuli like plosive speech sounds.

## Conclusion

Separable-affix verbs realized non-contiguously in sentences produce N400s. Furthermore the amplitude of these N400s varies as a continuous function of the incongruity between the stem and affix of the verb. Incongruity assessment with both corpus-based and behavioral rating measures performed well, although the behavioral measures produced more reliable correlations, likely as a result of reduced noise in comparison to corpus-based measures.

## Author Contributions

JH and FP contributed equally to the conception and design of the experiment, as well as writing the manuscript. JH analyzed the data.

## Conflict of Interest Statement

The authors declare that the research was conducted in the absence of any commercial or financial relationships that could be construed as a potential conflict of interest.

## Footnotes

1 www.audacityteam.org

2 http://corporafromtheweb.org/

3 http://github.com/jshanna100/ANOAR

## Appendix: Automated Non-Ocular Artifact Rejection (ANOAR)

The use of independent component analysis (ICA) or signal space projection (SSP) are powerful tools for the removal of ocular or cardiac artifacts (Haumann et al., 2016). Before the data can be transformed with these tools, however, it is important to remove bad channels and non-stationary noise from the signal, as these can significantly degrade their performance. Doing this by hand is tedious and time-intensive, and worse still, dependent on the judgments of individual researchers. Automated noise removal algorithms solve these problems, but have a disadvantage in that the same processes which remove non-stationary noise will typically also remove stationary noise such as ocular/cardiac artifacts – a task best left to ICA/SSP methods. The motivation of the ANOAR toolbox then is to remove non-stationary noise from ERP/F data, whilst leaving stationary noise intact.

With ANOAR, data are marked as bad in three possible ways: an entire channel, a particular trial on a particular channel, or a trial across all channels. Entirely bad channels are identified as follows: for a given channel, the continuous data are divided into sections of arbitrary length (e.g., 10 s), and for each section, the correlations of this channel with its neighbors are calculated. If the maximum correlation falls below a given threshold, then that time segment counts as a vote to mark the channel as bad. If the number of votes passes a given threshold, then the entire channel is marked as bad.

In order to identify bad channel/trial points, first the desired ERP/F average is computed, and then for each channel and each trial, the sum of square differences (SSDs) between ERP/F average and the individual channel/trial is calculated, across all time points in the trial. Therefore a given channel/trial combination will have a higher SSD the more it deviates from its channel’s ERP/F average. SSDs are arranged in a matrix with n rows and c columns, where n is the number of trials, and c is the number of channels (e.g., **Appendix Figure A1**, left). We could then mark any trial/channel point on the matrix as bad when it exceeds a given threshold, but at the moment, SSDs also partially reflect the stationary noise we want to retain. We remove the stationary noise by performing a linear regression of data in each channel/trial against the corresponding data from a given artifact signal (e.g., EOG, EKG, etc.), yielding an *r*-value which quantifies how much variance in the data are explained by the artifact signal. The SSDs are multiplied by (1-r), which decreases them to the extent that they are correlated with the artifact signal (e.g., **Appendix Figure A1**, middle).

Often a single trial will have aberrant data across many channels. In these cases it is usually best to remove the trial entirely. ANOAR allows this to be done when a given number of channel/trial points are marked as bad within a single trial.

Bad trials are removed from the data entirely, and bad channel/trial points are interpolated using the remaining good channels within that trial. Note that this approach reduces the dimensionality of the data. When, for example, ICA is performed later, the number of desired components should be specified to be somewhat less than the number of channels.

**FIGURE A1** Sum of square differences (SSDs) between trial-channels and their corresponding ERP average. Every point represents a channel-trial. The first matrix shows the unadjusted SSD. The second matrix shows the same SSD after being adjusted for EOG-related variance. Note the typical EOG noise – mostly present in the fronto-central channels located on the left and the middle columns of the matrix – is now mostly removed. The third matrix shows points of the data which are removed on account of too much non-ocular noise.
