# Supplementary figures and images for: Congruency of Separable Affix Verb Combinations Is Linearly Indexed by the N400
Source: Front Hum Neurosci. 2018 May 28;12:219. doi: 10.3389/fnhum.2018.00219 (PMC5985318; doi:10.3389/fnhum.2018.00219)

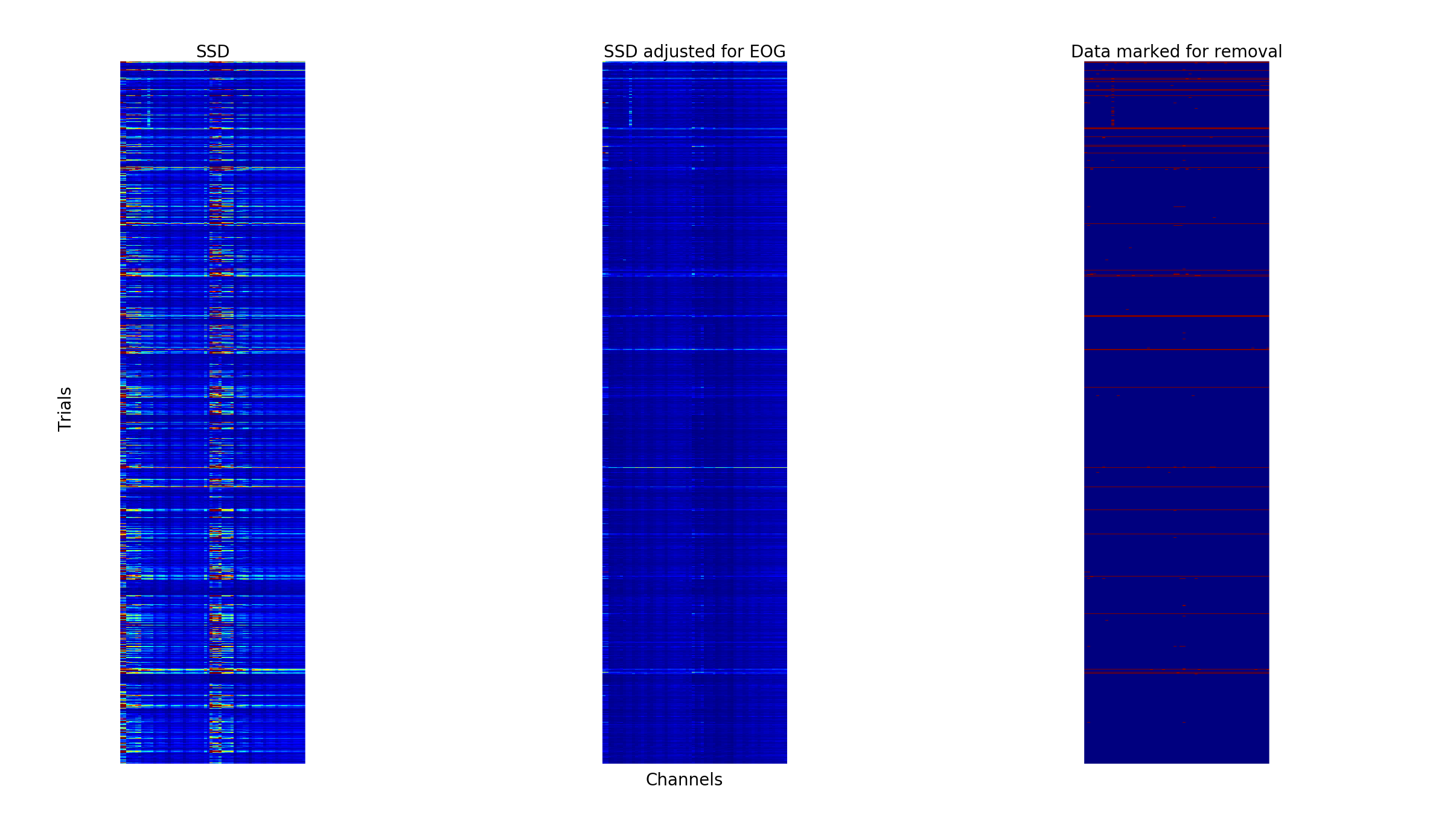

Supplement: Supplementary file 1 [file Image_1.TIF]
